# Transfer of weight information depends differently on used hand and handedness for perception and action

**DOI:** 10.1371/journal.pone.0278133

**Published:** 2022-12-13

**Authors:** Vonne van Polanen

**Affiliations:** 1 Movement Control and Neuroplasticity Research Group, Department of Movement Sciences, Biomedical Sciences Group, KU Leuven, Leuven, Belgium; 2 Leuven Brain Institute, KU Leuven, Leuven, Belgium; Justus Liebig Universitat Giessen, GERMANY

## Abstract

When lifting an object sequentially with the two hands, information about object weight can be transferred from one hand to the other. This information can be used to predictively scale fingertip forces and to form a perceptual estimation about the object’s weight. This study investigated how weight information can be transferred between the two hands and how this depends on the used hand and handedness of the participant. Right- and left-handed participants lifted light and heavy objects with the right or left hand in a pseudorandomized order and estimated the object’s weight. Results showed that predictive force scaling depended on the previously lifted object, with higher force rates if a previous object was heavy. This known effect of sensorimotor memory was mostly independent of the used hand and handedness, indicating that weight information could be transferred across hands for fingertip force scaling. Furthermore, a perceptual bias that depended on the previous lifted object was found, with lower weight estimations when the previous object was heavy compared to light. While this bias was found for both light and heavy objects in right-handers, it was only observed for light objects in left-handers. Notably, in contrast to the results on force scaling, this effect was not transferred across hands. These findings indicate that the transfer of weight information depends differently on the used hand and handedness for perceptual estimations and predictive force scaling.

## 1 Introduction

For skilful object lifting, fingertip forces must be predictively scaled towards the weight of the object. Predictive scaling is important, because feedback processes are often too slow and will result in less smooth movements [1, 2]. When the weight of the object is unknown, forces are typically based on the weight of the previously lifted object, an effect that has been attributed to sensorimotor memory [2–6]. Specifically, when a previous object is light, the force rates for the current lift are lower than if a previous object is heavy. These lower force rates, which can both be observed in vertical load forces and horizontal grip forces, indicate that predictive scaling is adjusted to the previous object weight.

Previous studies showed that this sensorimotor memory can be maintained for hours [7, 8] and the stored information can also be transferred between hands [9–12]. That is, when an object is lifted with one hand, the sensorimotor experience is used to scale forces for lifting an object with the other hand. The transfer of the sensorimotor memory for force scaling has generally been found not to depend on the used hand (i.e. right to left or left to right hand) and was equal in both directions [9, 10], although one study found larger effects when transferring from the right to the left hand in right-handed participants [12]. Furthermore, a more efficient transfer was found in right-handed compared to left-handed participants [10]. Differences between hands and handedness could suggest that processing of sensory information is lateralized to one hemisphere. However, since few studies on object lifting compared whether effects depended on the used hand or handedness, results on laterality and handedness remain scarce.

When lifting an object, the acquired sensory information can also be used to provide a perceptual judgement of the object’s weight. However, this estimate is not always veridical. Previous studies have shown that a previous lifted object can affect the estimated weight of the following lifted object [5, 6, 13]. When the lifted object is preceded by a heavy object, it is judged to be lighter than when preceded by a light object, indicating a perceptual bias. Previous lifts both affect the force scaling to object weight and weight perception of the next lift, although in opposite directions: after lifting a heavy weight, force rates are increased and weight perception is reduced. Nevertheless, it has been suggested that the perception and action processes are related. Since no perceptual bias was found when objects were placed on the hand instead of being lifted, the bias has been suggested to originate from the corrections to the fingertip forces, where the corrective reductions of the force would lead to a reduction in perceived weight and vice versa [5]. In support of this hypothesis, that study found a correlation between force and perceptual parameters. However, in a follow-up study, this correlation was not found [6], resulting in conflicting findings on the relation between force control and weight perception based on previous lifts.

If the sensory information about object weight used for force scaling and weight perception are related, it would be expected that this information is transferred across hands in a similar way for both processes as well. In contrast to the transfer of weight-related information for force scaling, less is known about the transfer of information for weight perception. A transfer of the perceptual bias, although weaker, was found from the left to the right hand [13], but this study was limited to a single trial. More research on the transfer of perceived weight has been performed in the context of the size-weight illusion. In this illusion, a small object is perceived to be heavier than a large object despite having the same mass [14]. It has been found that the illusion effect can be transferred from one hand to the other, although this effect was only visible with a transfer from the left to the right hand and not in the other direction [8]. However, this illusion has been shown to be independent from force scaling when looking at behaviour after repeated lifts [15, 16].

All in all, it remains unclear whether perceptual information about object weight can be transferred across hands and how this depends on the used hand and handedness of the participant. To address this issue, an experiment was performed where participants lifted objects of different weights. The perceptual bias based on a previous lifted object was used to examine whether weight information can be transferred from one hand to the other. Participants either lifted objects sequentially with the same hand or with different hands to test the ability to transfer sensory information. Force scaling and weight estimations were measured to investigate the sensorimotor memory of force scaling and the perceptual weight bias of previously lifted objects. In addition, it was measured whether the perceptual bias depended on the direction of transfer (from left to right, or from right to left) and on handedness (right-handed and left-handed participants).

The current study also serves to perform a replication of previous studies mentioned above that found effects of previous lifted objects on force scaling and perceptual weight estimation. Moreover, this study will also provide insight into the potential relation between the sensorimotor memory and perceptual biases from previous experience. If both processes are related to each other, it is expected they will depend similarly on the used hand and handedness and that effects correlate with each other. Since the sensorimotor memory can be transferred across hands [9, 10] and the perceptual bias was present in a transfer from the left to the right hand [13], it was expected that the perceptual bias would be transferred between hands as well.

## 2 Methods

### 2.1 Participants

Sixty participants were recruited for this study and divided over four groups. Handedness was assessed with the Edinburgh Handedness Inventory [17]. Twenty right-handed (mean LQ = 86) participants were assigned to the right-hand group (22.1 years, age range 18–27 years, 11 females) and another twenty right-handed participants (mean LQ = 88) were assigned to the left-hand group (21.8 years, 18–26 years, 9 females). Similarly, ten left-handed participants were placed in a right-hand (mean LQ = –85, 23.5 years, age range 19–31 years, 5 females) and another ten in a left-hand group (mean LQ = –86, 22.2 years, age range 20–27 years, 6 females). All participants provided written informed consent before participating. Ethical approval was obtained from the local ethical committee of KU Leuven.

### 2.2 Apparatus

**Fig 1:**
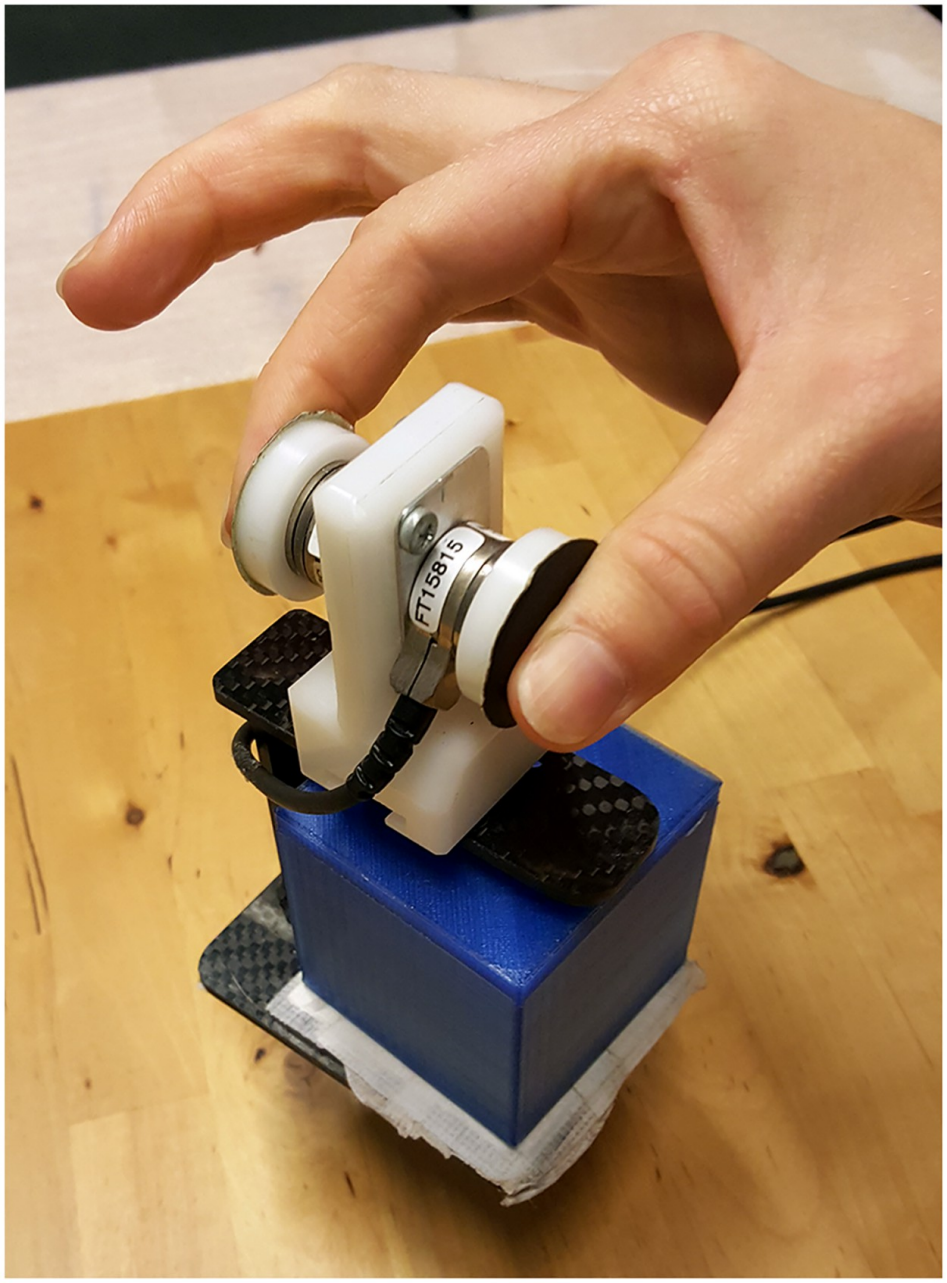
Experimental manipulandum with force sensors, covered with sandpaper. Different cubes could be placed in the manipulandum.

Two force sensors (Nano 17, ATI Industrial Automation) were attached to a manipulandum in which differently weighted objects could be placed (Fig 1). The force sensors were covered with sandpaper (No. P600) to increase the friction of the surface. Forces were sampled in three directions with a sample frequency of 1000 Hz.

Three 3D-printed objects were used in the experiment, which all measured 5 × 5 × 5 cm. They were filled with different amounts of lead shot to created weights of 105, 317 and 525 g (measured with a weighing scale). A fourth object of 260 g was used for practice trials. The manipulandum, including the force sensors, weighted approximately 120 g. Therefore, the lifted weights were 2.2, 4.3, 6.3 and 3.8 N, respectively, as measured by the force sensors. The cubes were placed under a paper cover to prevent visual cues.

To prevent participants seeing the experimenter changing the objects, participants wore PLATO glasses (Translucent Technologies) or a switchable screen (Magic Glass) was placed in front of them. The glasses and the screen could switch between an opaque and transparent state. Initially the PLATO glasses were used, but due to hygienic measures taken in response to the COVID-19 pandemic, a switchable screen was used for participants that took part in a later stage of the experiment. The function of both methods was similar and always a single method was used for a single participant.

### 2.3 Experimental procedure

Participants were seated at a table on which the manipulandum was placed and rested both hands on the table. They received an auditory cue indicating which hand should be used for the next trial. This cue was a computer voice saying ‘left’ or ‘right’. 1.5 s after this cue, the glasses or screen became transparent and participants reached for the manipulandum and lifted it with their thumb and index finger placed on the force sensors. They were told to lift the manipulandum straight, at a comfortable speed and to a height of ±5 cm and hold it steady. When the glasses or screen turned opaque again after 3 s, they replaced the manipulandum on the table again. Next, they were asked to estimate the weight of the lifted object on a self-chosen scale with no predetermined lower or upper limit (magnitude estimation). The experimenter then changed the object for the next trial. Before the experiment, 5 trials for each hand in a randomized order were performed with the practice object of 260 g to get familiar with the task procedure.

Trials were presented in a pseudo-randomized order of light and heavy weights, that were grasped with the right or left hand. There were four weight conditions where the order of the light (105 g) and heavy (525 g) objects were varied: light-light (LL), heavy-light (HL), light-heavy (LH) and heavy-heavy (HH). Here, the first weight refers to the object weight on the previous lift, and the second weight refers to the object weight of the current lift. For a trial, the object that was lifted was referred to as the current lift (n). The preceding lift (n-1) was the previous lift. For the next trial (n+1), the current lift (n) would become the previous lift, whereas the next lift (n+1) would be the current lift for that trial. For example, in a sequence of *LLHLHH*, an LL, LH, HL, LH and HH trial can be analysed.

In addition, the hand used in the previous and current lift was varied as well: either it was the same hand (S) or a different hand (D). Therefore, there were 8 conditions in the experiment: LLS, HLS, LHS, HHS, LLD, HLD, LHD, HHD. In the conditions where a different hand was used, the transfer of information from one hand to the other could be tested. The trials that were analysed differed across the four groups. For the two groups (right-handed and left-handed participants) in the right-hand groups, only trials where the current lift was performed with the right hand was analysed. Therefore, they would lift objects in the same hand conditions twice with their right hand, whereas in the different hand conditions they would lift the previous object with their left hand and the current one with their right hand. In contrast, for the two groups in the left-hand groups only trials where the current lift was performed with their left hand were included in the analysis. For those groups, the same hand conditions consisted of two lifts with the left hand and the different hand conditions were lifts first with the right hand followed by the left hand. Trials in which the current lift was performed with the other hand were discarded. In this way, the transfer towards the right hand and towards the left hand was examined in the right-hand and left-hand group, respectively. An overview of the conditions for each participant group is shown in Table 1.

**Table 1:** Overview of the participant groups and conditions. For each condition, the object weight in the previous and current trial (object order) is indicated (Light or Heavy). The used hands in the previous and current trial (hand order), where either the same (S) or different (D) hands are used, could be the left (L) or right (R) hand, dependent on the hand group.

		Conditions	LLS	HLS	LHS	HHS	LLD	HLD	LHD	HHD
		Object order	Light-Light	Heavy-Light	Light-Heavy	Heavy—Heavy	Light-Light	Heavy- Light	Light- Heavy	Heavy- Heavy
**Handedness**	**Hand group**	Hand order	Same	Same	Same	Same	Different	Different	Different	Different
Right	Right	Hand used	R-R	R-R	R-R	R-R	L-R	L-R	L-R	L-R
	Left	Hand used	L-L	L-L	L-L	L-L	R-L	R-L	R-L	R-L
Left	Right	Hand used	R-R	R-R	R-R	R-R	L-R	L-R	L-R	L-R
	Left	Hand used	L-L	L-L	L-L	L-L	R-L	R-L	R-L	R-L

The use of the right or left hand for the current lift were performed in two different participant groups to reduce the number of trials participants had to perform. If both conditions in which the left and the right hand were the current lift needed to be included, about twice as many trials would be needed which would have been demanding for the participants. In the present design, the right-hand group performed more trials with their right hand and left-hand trials were discarded, whereas the left-hand group performed more trials with their left hand and right-hand trials were discarded. Since only the effect of the previous lift on the current lift was of interest and the sensorimotor memory is known to be already established in a single lift [2, 4, 15], this imbalance in the use of hands should not have an effect on the results.

Each of the 8 conditions were presented 10 times, giving a total of 80 trials for analysis. To provide more variation in the weights used in the experiment, a third object of 317 g was used as a dummy object. This object was lifted in about 10% of the trials (15 trials), either with the left or right hand. These trials, and trials following dummy trials were not analysed. In addition, the sequence could also contain trials with the wrong hand order (e.g. left hand followed by right hand for the left-hand group; max 40 trials, this varied from sequence to sequence), which were also not analysed as explained above. Finally, the first trial was not preceded by any lift, so it was not analysed either. A total of 151 trials were presented of which at least 80 were entered into the analysis. The trial sequence was different for each participant.

### 2.4 Data analysis

Participants’ weight estimations were normalized to z-scores. Force data were filtered with a second order low-pass bidirectional Butterworth filter, with a cut-off frequency of 15 Hz. Load forces (LF) were the sum of the vertical forces and grip forces (GF) the average of the horizontal forces. These forces were differentiated with respect to time to obtain load forces rates (LFR) and grip force rates (GFR). From the force rates, the first peaks of the forces rates were of interest, since these reflect the planned force [1]. The first peaks (peakLFR1 and peakGFR1) were calculated between the time points of contact (GF>0.1 N) and just after lift-off (50 ms after lift-off, where LF equals object weight). To exclude peaks that were noise or accidental bumps when contacting the manipulandum, the first peak was used that was at least 30% of the maximum force peak as measured between these time points [6]. If all peaks before the maximum peak were lower than 30%, the maximum peak was used. Note that in some trials only one peak was present, which was automatically both the first and the maximum peak. Finally, the loading phase duration (LPD) was calculated as the time between onset of LF (LF>0.1 N) and lift-off (LF>object weight).

Trials in which the wrong object was placed, the wrong hand was used or the object was not lifted were removed from the perceptual analysis, as well as trials following these erroneous trials since the order would be wrong (total per group: 12, 0.7%, in right-handed with right hand; 8, 0.5%, in right-handed with left hand; 4, 0.5%, in left-handed with right hand; 3, 0.4%, for left-handed with left hand). In addition, trials in which the object was lifted twice, lifted before the screen became transparent and force measurement started, or pushed before lifting, were also removed from the force analysis (total per group: 19, 1.2%, in right-handed with right hand; 19, 1.1%, in right-handed with left hand; 8, 1%, in left-handed with right hand; 6, 0.7%, for left-handed with left hand).

### 2.5 Statistics

Statistical analyses were performed with JASP (version 0.16.3). The parameters of interest (perceptual answers, peakGFR1, peakLFR1, LPD) were averaged for each condition and in each group. A mixed analysis of variance (ANOVA) was performed with the following between-subject factors: handedness (2 levels: left-handed, right-handed), current hand (2 levels: left, right) and the following within-subject factors: previous hand (2 levels: same, different), previous weight (2 levels: light, heavy) and current weight (2 levels: light, heavy). The factor previous weight was of specific interest to test the perceptual bias and anticipatory force scaling. The factor current weight reflects potential differences between perception and force scaling to light and heavy objects. In addition, it was investigated how these weight effects would be influenced by the used hand and handedness of the participants. Three-way interactions were further analysed with separate mixed or repeated measures ANOVAs. Further post-hoc tests were performed with paired samples or independent t-tests with a Bonferroni correction. An alpha level of 0.05 was used.

Finally, it was tested whether the perceptual biases were related to the effects on the force parameters. The differences with respect to previous weight were used to investigate the relation between effects on perception and force. Similar to the z-scored perceptual answers, the peakGFR1 and peakLFR1 were converted to z-scores and the difference between a previous light and a previous heavy weight was calculated. The difference for the perceptual answers reflects the perceptual bias and the difference for the force parameter reflects the sensorimotor memory effect. A mixed model was used with the peakGFR1 or the peakLFR1 difference as predictor, in two separate analyses. The perceptual difference was the dependent variable and the peakGFR1 or peakLFR1 difference was entered as a covariate in a first-order autoregression model with maximum likelihood estimation. The same factors as in the mixed ANOVA (handedness, current hand, previous hand and current weight) were used in the model.

To test whether the parameters were related in general on a trial-by-trial basis, a similar mixed model was used where all trials of participants were entered. The trials were compared within participants, regardless of current used hand or previous weight. The peakGFR1 or peakLFR1 were entered as covariates, the perceptual answer as dependent variable and a first-order autoregression model with maximum likelihood estimation was used. As fixed factors, handedness and current hand were used, to distinguish between participant groups. This analysis was performed for current light and current heavy objects separately.

## 3 Results

In this study, participants lifted light and heavy objects and estimated their weight. It was investigated how participants planned their fingertip forces and estimated the weight of the current lift depending on the previous object they lifted. Participants performed the current lift with their right or left hand and were either right- or left-handed, resulting in four participants groups. Furthermore, the current lift was either performed with the same hand as the previous lift, or with a different hand to investigate whether information from the previous lift could be transferred to the other hand. All data used for analysis and figures and analysis scripts can be found on https://osf.io/kgrez/. The results for the mixed ANOVA for each variable are listed in Table 2.

**Table 2:** Results from the mixed ANOVA for the perceptual answers, first peak of grip force rate (peakGFR1), first peak of load force rate (peakLFR1) and loading phase duration (LPD). Effects are shown for within-subject effects previous hand (PH), current hand (CH) and previous weight (PW) and between-subject effects current weight (CW) and handedness (H). Significant effects are shown in bold.

	*Perceptual answers*			*peakGFR1*			*peakLFR1*			*LPD*		
*Effect*	*F(1*,*56)*	*p*	*η*_*p*_^*2*^	*F(1*,*56)*	*p*	*η*_*p*_^*2*^	*F(1*,*56)*	*p*	*η*_*p*_^*2*^	*F(1*,*56)*	*p*	*η*_*p*_^*2*^
PH	1.70	0.20	0.03	0.56	0.46	0.01	**5.30**	**0.03**	**0.09**	0.00	0.96	0.00
CW	**21989**	**<0.001**	**1.00**	**20.15**	**<0.001**	**0.27**	**46.60**	**<0.001**	**0.45**	**507.19**	**<0.001**	**0.90**
PW	**23.81**	**<0.001**	**0.30**	**158.07**	**<0.001**	**0.74**	**92.49**	**<0.001**	**0.62**	**207.19**	**<0.001**	**0.79**
H	**5.78**	**0.02**	**0.09**	0.01	0.92	0.00	0.01	0.92	0.00	0.08	0.78	0.00
CH	**7.52**	**0.01**	**0.12**	0.81	0.37	0.01	0.00	0.98	0.00	0.39	0.53	0.01
PH*H	0.05	0.82	0.00	1.04	0.31	0.02	0.03	0.86	0.00	1.05	0.31	0.02
PH*CH	0.11	0.74	0.00	**5.39**	**0.02**	**0.09**	2.42	0.13	0.04	0.35	0.56	0.01
CW*H	0.30	0.59	0.01	1.98	0.17	0.03	0.60	0.44	0.01	0.00	0.99	0.00
CW*CH	0.91	0.34	0.02	1.62	0.21	0.03	0.05	0.82	0.00	0.47	0.50	0.01
PW*H	0.55	0.46	0.01	0.11	0.74	0.00	0.07	0.80	0.00	0.55	0.46	0.01
PW*CH	1.16	0.29	0.02	1.04	0.31	0.02	0.04	0.84	0.00	0.00	0.99	0.00
PH*CW	0.71	0.40	0.01	0.69	0.41	0.01	0.37	0.54	0.01	0.12	0.73	0.00
PH*PW	**10.05**	**<0.001**	**0.15**	**30.96**	**<0.001**	**0.36**	**11.45**	**<0.001**	**0.17**	**45.23**	**<0.001**	**0.45**
CW*PW	**15.38**	**<0.001**	**0.22**	0.54	0.47	0.01	2.24	0.14	0.04	**64.73**	**<0.001**	**0.54**
H*CH	0.02	0.89	0.00	0.70	0.41	0.01	0.01	0.93	0.00	0.03	0.86	0.00
CW*H*CH	0.89	0.35	0.02	**5.61**	**0.02**	**0.09**	0.29	0.59	0.01	0.48	0.49	0.01
PW*H*CH	0.41	0.52	0.01	0.04	0.85	0.00	1.43	0.24	0.03	0.13	0.72	0.00
PH*CW*H	0.00	0.99	0.00	0.53	0.47	0.01	0.79	0.38	0.01	0.66	0.42	0.01
PH*CW*CH	0.98	0.33	0.02	3.67	0.06	0.06	1.34	0.25	0.02	0.43	0.52	0.01
PH*CW*H*CH	0.60	0.44	0.01	0.60	0.44	0.01	0.04	0.85	0.00	0.22	0.64	0.00
PH*PW*H	0.81	0.37	0.01	0.21	0.65	0.00	1.19	0.28	0.02	0.65	0.42	0.01
PH*PW*CH	2.03	0.16	0.04	0.42	0.52	0.01	0.08	0.77	0.00	3.68	0.06	0.06
CW*PW*H	**8.65**	**0.01**	**0.13**	0.07	0.80	0.00	0.01	0.95	0.00	0.55	0.46	0.01
CW*PW*CH	0.90	0.35	0.02	2.08	0.16	0.04	0.79	0.38	0.01	0.00	1.00	0.00
CW*PW*H*CH	0.03	0.86	0.00	1.54	0.22	0.03	0.10	0.76	0.00	0.01	0.93	0.00
PH*CW*PW	3.00	0.09	0.05	0.71	0.40	0.01	0.99	0.33	0.02	**10.02**	**<0.001**	**0.15**
PH*CW*PW*H	0.09	0.77	0.00	1.80	0.19	0.03	0.25	0.62	0.00	0.18	0.68	0.00
PH*CW*PW*CH	3.00	0.09	0.05	1.79	0.19	0.03	3.13	0.08	0.05	0.00	1.00	0.00
PH*CW*PW*H*CH	0.00	0.98	0.00	0.41	0.53	0.01	2.24	0.14	0.04	0.85	0.36	0.02

### 3.1 Weight perception depends on the used hand and handedness

**Fig 2:**
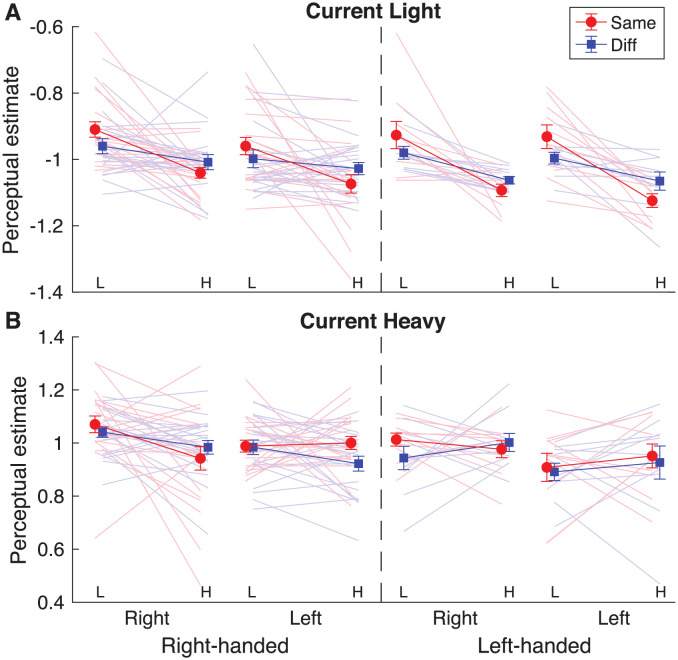
Results for the perceptual estimates in each condition for current light (**A**) and current heavy (**B**) objects. Lines are shown from a previous light (L) to a previous heavy (H) lift for each participant group, who were right-handed (before dashed line) or left-handed (after dashed line) and used their right or left hand in the current lift. In the previous lift, the same hand (red, Same) or a different hand (blue, Diff) was used. Solid lines and error bars represent means and standard errors. Light shaded lines indicate individual participants. With a perceptual bias in the expected direction, a decreasing line from L to H is expected. This difference was only significant when using the same (red lines), not different (blue lines) conditions. For right-handers this was the case for both current light (A) and heavy objects (B), but for left-handers only for current light (A) objects.

Participants estimated the weight of each object after they had lifted it. Especially the effect of previous weight was of interest, which would indicate the presence of a perceptual bias. Results for the perceptual estimates are shown in Fig 2.

The mixed ANOVA indicated significant effects of current weight (F(1,56) = 21989.0, p<0.001, *η*_p_^2^ = 1.00), previous weight (F(1,56) = 23.8, p<0.001, *η*_p_^2^ = 0.30) and handedness (F(1,56) = 5.8, p = 0.020, *η*_p_^2^ = 0.09) (Table 2). However, there were also interactions between current weight × previous weight (F(1,56) = 15.4, p<0.001, *η*_p_^2^ = 0.22) and a three-way interaction between current weight × previous weight × handedness (F(1,56) = 8.7, p = 0.005, *η*_p_^2^ = 0.13). Therefore, these results will be interpreted in light of the three-way interaction.

To further analyse this three-way interaction, two separate repeated measures ANOVAs were performed for each handedness group, with the within factors current weight and previous weight. In the right-handed group, an effect of current weight was found (F = 15970.95 p<0.001, *η*_p_^2^ = 0.998). As expected, it was found that a light object was perceived to be lighter than a heavier object. In addition, an effect of previous weight (F = 20.03, p<0.001, *η*_p_^2^ = 0.339) was found, which indicated a perceptual bias: when previously a heavy object was lifted, the current object was perceived to be lighter than if the previous object was light. This is seen in Fig 2, where the lines decrease from a previous light (L) to a previous heavy (H) object.

For the left-handed group, a similar effect of current weight (F = 10.009, p = 0.005, eta = 0.345) also showed that the light object was perceived to be lighter than a heavy object. For this group, also a main effect of previous weight was found (F = 9266.16, p<0.001, *η*_p_^2^ = 0.998), but also an interaction between previous weight × current weight (F = 22.787, p<0.001, *η*_p_^2^ = 0.545). This interaction indicated that there was only a perceptual bias when the current lifted object was light (p<0.001), but not when this object was heavy (p = 1.00). In Fig 2, this can be observed as decreasing lines from L to H for current light objects, but relatively flat lines for current heavy objects.

Furthermore, the overall mixed ANOVA showed an interaction between previous hand × previous weight (F(1,56) = 10.1, p = 0.002, *η*_p_^2^ = 0.15). Post-hoc tests indicated that the perceptual bias was only seen when objects were lifted with the same (p<0.001), but not with different hands (p = 0.244). In Fig 2, this can be seen as decreasing lines for the same hand (red) and relatively flat lines for different hands (blue). When using the same hand, the object was estimated heavier than when using a different hand, in the case when the previous object was light (p<0.001). No difference between hands was observed when the previous object was heavy (p = 1.00). Importantly, these results indicate that no transfer of the perceptual bias was found, but it was only seen when lifting objects with the same hand, independent of handedness.

Finally, there was also a main effect of current hand (F(1,56) = 7.5, p = 0.008, *η*_p_^2^ = 0.12). This effect indicated that an object that was lifted with the right hand was estimated to be heavier than when lifted with the left hand.

Overall, these results indicated that a perceptual bias was found, but this could not be transferred between hands. In addition, whereas the perceptual bias was seen for both light and heavy objects in right-handers, it was only found in light objects for left-handers. Finally, an object felt heavier when it was lifted with the right hand, regardless of handedness.

### 3.2 Peak grip force rates: Effects of previous object depend on the used hand

To examine the force scaling for the lifted object, the first peak of the force rate was calculated. Next, the different participant groups were compared with a mixed ANOVA (Table 2).

**Fig 3:**
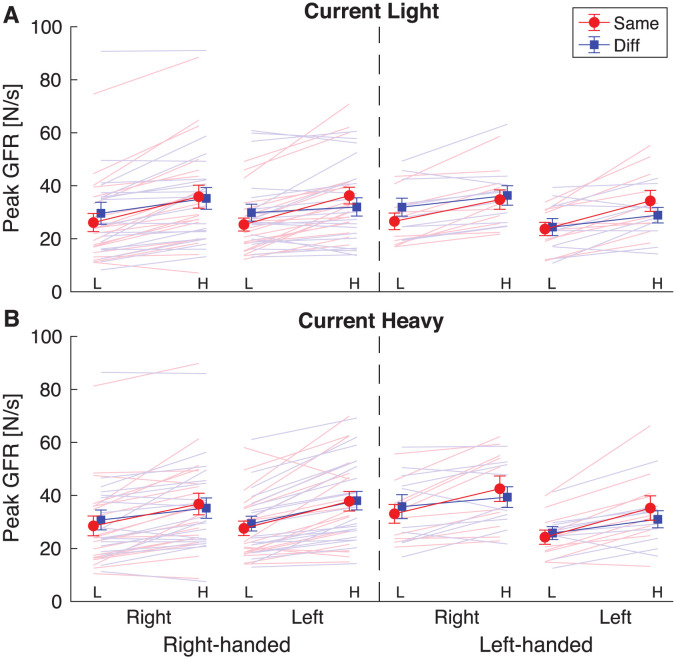
Results for the first peak grip force rate (peak GFR) in each condition, for current light (**A**) and current heavy (**B**) objects. Lines are shown from a previous light (L) to a previous heavy (H) lift for each participant group, who were right-handed (before dashed line) or left-handed (after dashed line) and used their right or left hand in the current lift. In the previous lift, the same hand (red, Same) or a different hand (blue, Diff) was used. Solid lines and error bars represent means and standard errors. Light shaded lines indicate individual participants. If force scaling is adjusted to the previous weight, and increasing line from L to H is expected. This was the case in all conditions.

The peakGFR1 results are illustrated in Fig 3. For the peakGFR1, a significant main effect of current weight (F(1,56) = 20.1, p<0.001, *η*_p_^2^ = 0.270) was found, but this effect will be discussed in light of the significant three-way interaction between current weight × handedness × current hand (F(1,56) = 5.6, p = 0.021, *η*_p_^2^ = 0.09). To further analyse this interaction, two mixed ANOVAs were performed for the right-handed and left-handed group, with the factors current weight and current hand. For both groups, only a main effect of current weight was found (right-handers: F = 8.745, p = 0.005, *η*_p_^2^ = 0.187; left-handers: F = 9.366, p = 0.007, *η*_p_^2^ = 0.342). The grip force rate was larger when lifting heavy compared to light objects and this appeared to be the case in all conditions. When the values were observed in order to explain the interaction, it seemed that the difference between light and heavy objects was larger for right-handers when lifting with their left hand, compared to their right hand, whereas this was reversed for the left-handers. However, no significant effects of current hand were found in these post-hoc tests.

Secondly, the initial mixed ANOVA also demonstrated a significant effect of previous weight (F(1,56) = 158.1, p<0.001, *η*_p_^2^ = 0.74). This effect needs to be interpreted in light of the interaction between previous hand × previous weight (F(1,56) = 31.0, p<0.001, *η*_p_^2^ = 0.36). Post-hoc tests indicated that when the previous object was light, a lower peakGFR1 was used than when the previous object was heavy, indicating an effect of sensorimotor memory. This is shown in Fig 3 as increasing lines from previous light (L) to previous heavy (H) objects. This difference was significant both when the same (p<0.001) and different hands (p<0.001) were used (red and blue lines in Fig 3, respectively). However, the difference was somewhat smaller for different than same hands, resulting in opposite effects for previous light (peakGFR1 smaller in same compared to different hands, p<0.001) and previous heavy (peakGFR1 larger in same compared to different hands, p = 0.003).

Finally, there was an interaction between previous hand × current hand (F(1,56) = 5.4, p = 0.024, *η*_p_^2^ = 0.09), but there were no significant post-hoc tests (all ps>0.206).

To summarize, the grip force rates depended on the previous lifted weight: a higher force rate was seen when the previous object was heavy compared to light, demonstrating an effect of sensorimotor memory. This effect was both seen when using the same as when using different hands, indicating that it could be transferred across hands, although it appeared to be less strong when transferring between hands. Furthermore, the grip force rate was higher when lifting heavy compared to light objects. A three-way interaction suggested this depended on the used current hand and handedness, but post-hoc tests provided no further significant effects of these factors.

### 3.3 Peak load force rates: Effects of previous object are independent of used hand and handedness

**Fig 4:**
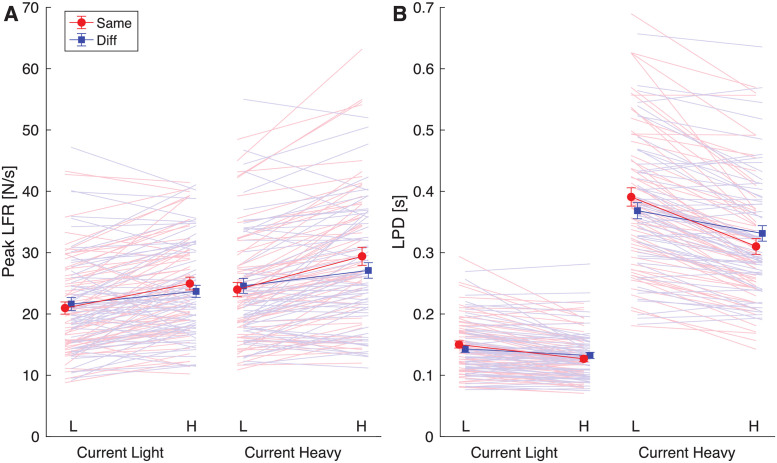
Results for the first peak load force rate (peak LFR, **A**) and loading phase duration (LPD, **B**). Lines are shown from a previous light (L) to a previous heavy (H) lift pooled over all participant groups (right- and left-handed, using right and left hand in current lift). Solid lines and error bars represent means and standard errors. Light shaded lines indicate individual participants. If force scaling is adjusted to the previous weight, an increasing line from L to H is expected for the peakLFR and a decreasing line for LPD. This was the case in all conditions.

For the peak load force rates, the participant groups were also compared in a mixed ANOVA (Table 2). The peakLFR1 did not depend on the handedness or current used hand in the task. Therefore, the groups are shown pooled in Fig 4A.

The mixed ANOVA showed a significant effect of previous weight (F(1,56) = 92.5, p<0.001, *η*_p_^2^ = 0.62), which indicated that the peakLFR1 was higher when the previous object was heavy compared to light (increasing lines from L to H in Fig 4A). There was also a main effect of current weight (F(1,56) = 46.6, p<0.001, *η*_p_^2^ = 0.45) that demonstrated that the peakLFR1 was higher when the current object was heavy compared to light. Together, these effects indicate that the load force rate was higher when lifting heavy objects, but also when previously lifting a heavy object, indicating an effect of sensorimotor memory.

There was also a main effect of previous hand (F(1,56) = 5.3, p = 0.025, *η*_p_^2^ = 0.09), but this needs to be interpreted in light of the interaction between previous hand × previous weight (F(1,56) = 11.5, p = 0.001, *η*_p_^2^ = 0.17). When using the same hand, the force rate was higher than when using different hands, but this difference was only seen when the previous object was heavy (p<0.001), not light (p = 0.24). The effect of sensorimotor memory was seen both when using the same or different hands (p<0.001).

### 3.4 Loading phase duration: Effects of previous object are independent of used hand and handedness

The LPD was not different between the different participant groups, as the mixed ANOVA showed no significant effects or interactions with the between factors (Table 2). Therefore, results are shown pooled across all participants (Fig 4B). There were main effects of previous weight (F(1,56) = 207.2, p<0.001, *η*_p_^2^ = 0.79) and current weight (F(1,56) = 507.2, p<0.001, *η*_p_^2^ = 0.90), as well as interactions between previous hand × previous weight (F(1,56) = 45.2, p<0.001, *η*_p_^2^ = 0.45), current weight × previous weight (F(1,56) = 64.7, p<0.001, *η*_p_^2^ = 0.54), but these need to be discussed in light of the 3-way interaction of previous hand × current weight × previous weight (F(1,56) = 10.0, p = 0.003, *η*_p_^2^ = 0.15).

The difference between light and heavy objects were significant in the post-hoc tests for all conditions (all p<0.001). Similarly, differences between previous heavy and previous light objects were significant in all post-hoc tests (all p<0.02). When the previous object was heavy, the LPD was shorter than when the previous object was light and the LPD was longer when objects were heavy compared to light (decreasing lines from L to H in Fig 4B). The difference between using the same hand and different hands was only significant when the object was heavy (LH and HH conditions, both p<0.008), but not when the object was light (p>0.26). Here, the LPD was shorter when the same hands were used compared to different hands.

All in all, the peakLFR1 and the LPD depended on the weight of the current object and the previous object. There did not seem to be any differences between the hand that was used or the hand preference of the participant, since these effects were found in all conditions.

### 3.5 Relation between perceptual biases and force parameters

**Fig 5:**
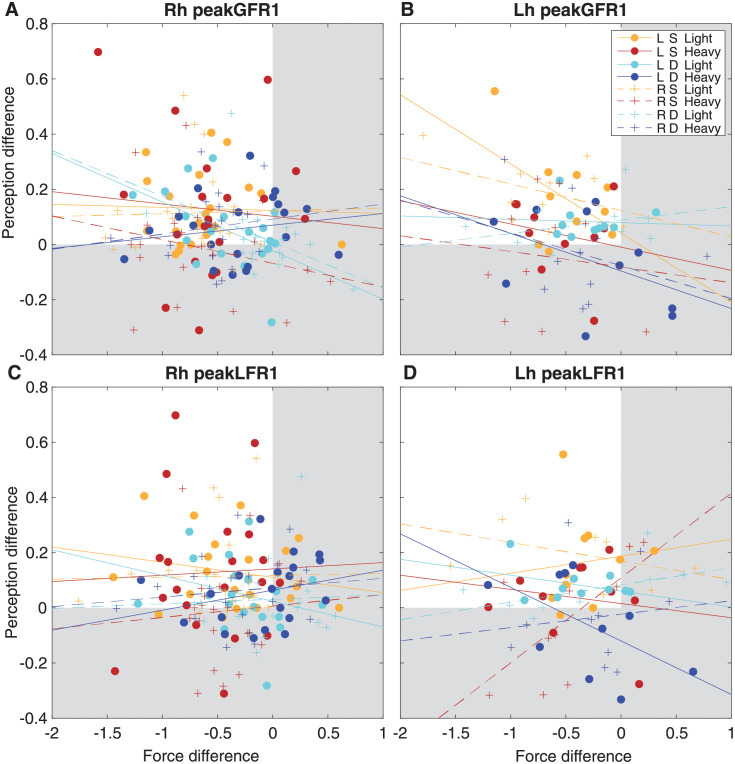
Correlations between perceptual bias and force scaling difference. The difference was calculated between a previous heavy and a previous light object, for the first peak of grip force rates (peakGFR1, **A-B**) and load force rates (peakLFR1, **C-D**). Data are shown for each participant group (**A,C** Rh = right-handed, **B,D** Lh = left-handed), and the use of the left hand (L, circles) or right hand (R, plusses). Colours indicate the use of the same (S) or different (D) for a current light or heavy object. Lines indicate the regression lines and points represent individual participants. Values within the white square indicate points in the expected direction (perceptual bias and sensorimotor memory). Note that none of the correlations were significant after Bonferroni correction.

The results described above show effects of a previous lifted object on perceptual answers (perceptual bias) and force scaling (sensorimotor memory). Individual participant data for all conditions are shown in Fig 5 as differences between a previous heavy and a previous light lift. Here, positive values on the y-axis (perception) indicate a perceptual bias in the expected direction and negative values on the x-axis (force scaling) indicate the expected effect from the sensorimotor memory. When values fall within the white square, both differences are in the expected direction. In line with the results as described above, this is mainly the case for lifting light objects and when lifting with the same hand.

To investigate the relation between effects of previous object on the perceptual answers and the force scaling, a mixed model was performed on the differences between a previous light and a previous heavy object. In the mixed model using the peakGFR1 as predictor, an effect of peakGFR1 was found (F(1,231) = 8.3, p = 0.004), in addition to an interaction of handedness × previous hand × current weight × peakGFR1 (F(1,223) = 6.8, p = 0.010). This suggest that the effects of the previous weight on the grip force rate seem to be related to the perceptual biases. Finally, a main effect of current weight was also found (F(1,231) = 5.8, p = 0.017), but this effect was already discussed for the ANOVA results.

For the peak load force rate, interactions of current hand × peakLFR1 (F(1,238) = 4.4, p = 0.038) and of handedness × current hand × current weight × peakLFR1 (F(1,235) = 5.5, p = 0.020) were found. This suggests that the peakLFR1 difference was related to the perceptual bias, although this depended on the specific condition. Finally, other effects of previous hand (F(1,201) = 12.0, p = 0.001), current weight (F(1,211) = 6.9, p = 0.009) and an interaction between handedness × current weight (F(1,211) = 5.9, p = 0.016) were found. Since effects of these factors were already discussed in the results of the ANOVA, they will not be further discussed here.

Since the peak force rates also interacted with some conditions, separate correlations were performed between the peak force differences and the perceptual biases in each condition (2 handedness × 2 current hand × 2 previous hand × 2 current weight = 16 correlations for peakGFR1 and for peakLFR1). The correlations are illustrated in Fig 5. However, after Bonferroni correction, none of these correlations were significant (p>0.32).

For the trial-by-trial analysis, it was found that peakGFR1 was a significant predictor for the perceptual estimate, both for light (F(1,2447) = 55.0, p<0.001) and heavy (F(1,2477) = 5.3, p = 0.022) objects. PeakLFR1 was only a significant predictor in light (F(1,2436) = 30.9, p<0.001), but not for heavy objects (F(1,2476) = 2.9, p = 0.086). This suggests that on a trial-by-trial basis, perceptual estimates were related to the grip force rate peak and also to the load force rate peak, but for the latter parameter only when lifting light objects.

## 4 Discussion

The main aim of this study was to investigate how weight information can be transferred between the hands when lifting objects. Specifically, the effects of the used hand and handedness on force scaling and the perceptual bias that occurs in weight perception depending on the previously lifted object were examined. Four groups of participants who were either right- or left-handed lifted objects of different weights and used their left or right hand to lift these objects. They sequentially lifted two objects with the same hand or with different hands and estimated the objects’ weight. It was found that the previously lifted weight affected anticipatory force scaling and that information from the previous lifted weight could be transferred across hands for predictive force scaling. However, while a perceptual bias was found when using the same hands, particularly for light objects, this information could not be transferred to the other hand.

When an object was lifted after a light object, it was perceived to be heavier than when it was lifted after a heavy object, indicating that a perceptual bias was present. This finding replicates results from previous studies in right-handed participants [5, 6, 13] and extends it to left-handed participants. While there are few studies on the effect of handedness on weight perception, no apparent differences in weight discrimination are known between right- and left-handers [18–20], but the size-weight illusion seems to differ between hands for right-handed and not for left-handed participants [10]. The present study shows that a perceptual weight bias from previous lifted objects is present in both right and left-handed participants.

While the perceptual bias was found both when lifting light and heavy objects for right-handers, it was only observed with light objects for left-handers. In a previous study with right-handed participants, it was also found that the perceptual bias was only seen for light objects, not heavy [5]. In another study, both for light and heavy objects a bias was found, but the effect seemed smaller for the heavy objects [6]. In both these studies only right-handed participants using their right hand to lift the objects were included. Possibly the small effect seen for heavy object relates to the relative discrimination performance depending on the weight of the object. That is, the weight difference that can be perceived needs to be larger for heavy compared to light objects. A small perceptual bias would not be large enough to be discriminated and object weights would be judged similarly.

In line with this explanation, it is possible that the presence of the perceptual bias depends on the sensitivity to weight in a specific condition. For instance, weight discrimination is worse for passive perception, when objects are placed in the hand, compared to active lifting [21], which might explain why only a perceptual bias was found for active lifting in [5]. On the other hand, if the perceptual bias is related to weight discrimination, one would expect a smaller or absent bias in the non-dominant hand, because weight discrimination is worse for this hand [18]. However, in the present study, the perceptual bias did not seem to depend on the hand that was used. In addition, the present study found only effects in light objects, not heavy, for the left-handed participants, while this was found for both objects in right-handers, but there is no evidence that left-handers are better or worse at weight discrimination than right-handers [18–20]. Future research could further explore the relation between the perceptual weight bias and weight discrimination by testing these parameters in the same group of participants.

In accordance with previous research, the previous lifted object also affected the force scaling for the current lift [2–6]. The forces were scaled higher and as a consequence the loading phase duration was shorter after lifting a heavy object compared to after lifting a light object. For the load force and loading phase duration, no effects of hand or handedness were found. Although there were effects for the grip force rate dependent on the hand used or the handedness of the participant, no significant differences were found in the post-hoc tests. An interaction between previous hand and previous weight suggested that the transfer of sensorimotor memory between hands was somewhat weaker than the sensorimotor memory effect in the same hand, but it was present in both cases. Therefore, there seemed to be no apparent differences between the force parameters.

Altogether, while both for perceptual estimation and anticipatory force scaling an effect of the previous lifted object was found, this information could only be transferred across hands for force scaling, not for perceptual estimation. This suggests that there are differences in the transfer of weight information for object lifting and object perception. Furthermore, although sensorimotor memory effects in peak grip force and peak load force rate were significant predictors of perceptual biases, no significant correlations were found when looking at conditions separately. Previous studies also found conflicting results concerning the correlation between force and perceptual parameters [5, 6]. Van Polanen and Davare [5] proposed that corrections made to the planned forces altered the perceived weight, since the perceptual bias was only found when participants actively lifted the object, not when it was placed on their hand. However, the present results indicate that when using different hands, these corrections to force scaling take place but no difference in weight is perceived. While force parameters and perceptual estimates were still related on a trial-by-trial basis, the differences in findings on the transfer of information questions the hypothesis that these processes are related. Earlier research on the size-weight illusion showed that size information differently affects weight estimation and anticipatory force scaling, especially after repeated lifting [15, 16]. The present research indicates that previous experience differently affects weight estimation and force scaling when information needs to be transferred between hands. Weight information might be stored in different ways for perception and action, so it cannot be retrieved equally well for the other hand, or the information might be transferred differently between hemispheres.

Another finding was that the weight perception differed between lifting an object with the right and the left hand, independent of handedness. Objects that were lifted with the right hand felt heavier than objects lifted with the left hand. Since this effect was not different for left- and right-handers, this does not seem to be an effect of hand dominance. Previous research found differences in weight discrimination between hands, but also between dominant and non-dominant hands [18], whereas it also seemed to depend on left or right dominance in hearing [19]. However, no previous literature is known that compared weight estimations between right and left hands in both right- and left-handed participants. A possible explanation for a bias between the right and left hand might be that in daily life, the right hand is used more often. This is also the case for left-handers, since the world is oriented towards right-handed persons. However, it is unclear how this would result in an weight overestimation for the right hand.

In contrast to this finding for weight perception, there were no apparent differences in force scaling for the used hand or handedness of the participant. For the grip force, there were interaction effects of current weight with the current hand, but this also interacted with handedness. The post-hoc tests did not reveal significant results, but because of the interaction with both current hand and handedness, this finding seems more likely to result from hand dominance, where force scaling might be more precise for the dominant hand. The dynamic dominance hypothesis [22, 23] suggests that the dominant and non-dominant hand have specific roles in motor control and predicts more feedforward control in the dominant hemisphere. However, because no significant post-hoc effects were found for the grip force, and no effects of hand dominance seemed to be present for the load force and the loading phase duration, the current results do not provide strong support for a dependence of hand dominance. All in all, these contradictory results demand more research on the role of handedness and used hand in anticipatory force scaling for object lifting and the transfer of sensory information.

In conclusion, the present findings indicate that the sensorimotor memory from lifting an object can be transferred between hands, both in right- and left-handed participants. In contrast, a perceptual bias from lifting a previous object was only seen when using the same hand, both for right- and left-handers, although only with light objects for the latter group. These results provide insight into how information about object weight is stored and transferred between hemispheres to be used for lifting objects and perceiving their weight. These processes seem to depend differently on the used hand and handedness for perception and action.
